# Clinical and mutational characteristics of oculocutaneous albinism type 7

**DOI:** 10.1038/s41598-024-57969-0

**Published:** 2024-03-30

**Authors:** C. C. Kruijt, G. C. de Wit, H. M. van Minderhout, N. E. Schalij-Delfos, M. M. van Genderen

**Affiliations:** 1 Bartiméus Diagnostic Center for Complex Visual Disorders, Zeist, The Netherlands; 2 Department of Ophthalmology, Leiden University Medical Center, J3-S, Albinusdreef 2, P.O. Box 9600, 2300 RC Leiden, The Netherlands; 3 Department of Ophthalmology, Medical Center Haaglanden, The Hague, The Netherlands; 4 Department of Ophthalmology, University Medical Center Utrecht, Utrecht, The Netherlands

**Keywords:** Eye manifestations, Eye diseases

## Abstract

The purpose of this paper is to expand on the phenotype of oculocutaneous albinism type 7 (OCA7). We described three patients with OCA7: two from a consanguineous family of Kurdish origin and one patient of Dutch origin. We compared them with all patients described to date in the literature. All newly described patients had severely reduced visual acuity (VA), nystagmus, hypopigmentation of the fundus, severe foveal hypoplasia, and chiasmal misrouting. None had iris translucency. All patients had normal pigmentation of skin and hair. We found one novel mutation in the Dutch patient: c.565G > A; p.(Gly189Ser)*.* We compared our patients to the 15 described in the literature to date. All 18 patients had substantially pigmented skin and hair, very poor VA (0.4–1.3 logMAR), nystagmus, (mild) ocular hypopigmentation, foveal hypoplasia, and misrouting. Although pigmentation levels were mildly affected in OCA7, patients had a severe ocular phenotype with VA at the poorer end of the albinism spectrum, severe foveal hypoplasia, and chiasmal misrouting. OCA7 patients had a phenotype restricted to the eyes, and similar to that of X-linked ocular albinism. We therefore propose to rename the disorder in ocular albinism type 2. Unfolding the role of *LRMDA* in OCA7, may bring us a step closer in identifying the responsible factors for the co-occurrence of foveal hypoplasia and misrouting.

## Introduction

**Figure 1:**
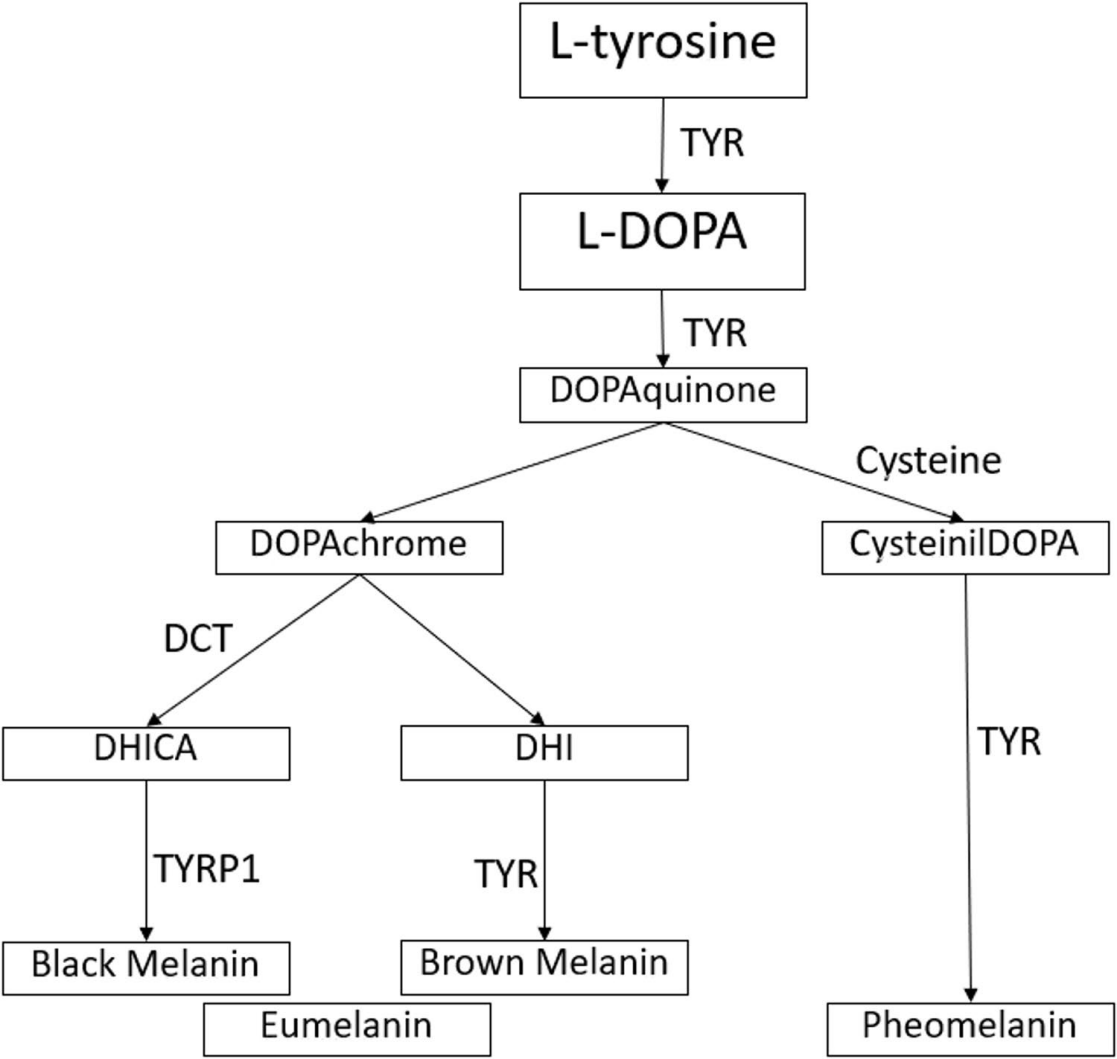
Schematic presentation of the synthesis of eumelanin and pheomelanin in the melanosome.

Clinical characteristics of albinism include reduced visual acuity (VA), nystagmus, iris translucency, hypopigmentation of the retina, foveal hypoplasia, and misrouting of the visual pathways. In autosomal recessively inherited oculocutaneous albinism (OCA) pigmentation of skin and hair is usually affected, but in X-linked ocular albinism (OA1) hypopigmentation is restricted to the eyes^1^. Oculocutaneous albinism type seven (OCA7) is one of nine known non-syndromic types of albinism (OCA1-8 and OA1), and is one of the rarest forms of non-syndromic albinism. In 2013 Grønskov and co-workers were the first to identify the *LRMDA gene*, then known as *c10orf11* gene, through homozygosity mapping in a consanguineous Faroese family with albinism^2^. The *LRMDA* gene is located on chromosome 10 in the 10q22.2-q.22.3 region (OCA7; OMIM #615179) and encodes a 198 amino acid protein containing three leucine-rich repeats (LRRs) and one LRR C-terminal domain. The family of LRRs-containing proteins encompasses members with a variety of functions, including cell adhesion and signaling, neuronal development, and RNA processing^3^. Knockdown of the LRMDA zebrafish homolog resulted in a reduction in pigmentation, the number of pigmented melanocytes, dopachrome tautomerase (DCT), and the number of melanoblasts. DCT is an important enzyme in the melanin synthesis pathway^2^. Without DCT, dopachrome is not converted to dihydroxyindole carboxylic acid (DHICA). Part of the eumelanin synthesis pathway is blocked, and dopachrome spontaneously changes into dihydroxyindole (DHI). Under the influence of tyrosinase, eumelanin can still be produced (Fig. 1)^4^. This may be the reason that, although *LRMDA* is important for the development and differentiation of melanocytes, mutations do not result in complete lack of pigmentation in zebrafish nor humans^2^. The OCA7 protein is localized on the limiting membrane of melanosomes. Melanosomes can be in different stages, depending on the maturation. Stage I melanosomes contain premelanosomes protein (PMEL), and an amyloid sequestering protein, apolipoprotein E, that is localized on the surface of melanosome intraluminal vesicles. PMEL associates with the intraluminal vesicles, and forms functional amyloid fibrils. In stage II melanosomes PMEL fibrils are mature. Then, melanin synthesis and pigment deposition starts in stage III and is completed in stage IV. Beyers et al. demonstrated that the PMEL protein is abnormally processed and less mature fibrils are present in melanosomes of OCA7 knock-out (OCA7-KO) in newly generated MNT1 melanocytes. In OCA-KO cells an abundance of stage I and aberrant melanosomes were present, and significantly less stage II and III melanosomes than in wildtype cells. Also, pH-levels are lower in the melanosomes of these cells. Lower pH-levels inhibit tyrosinase activity, leading to a reduction in melanin synthesis^5,6^. Thus, both maturation of melanosomes and melanin synthesis are affected in OCA7. Melanin synthesis may be affected by lower dopachrome tautomerase levels, and a lower pH-level in the melanosome^2,5^.

In this study, we describe our cohort of OCA7 patients in the Netherlands, and compare the phenotype to that of all patients with OCA7 described in the literature.

## Patients and methods

**Figure 2:**
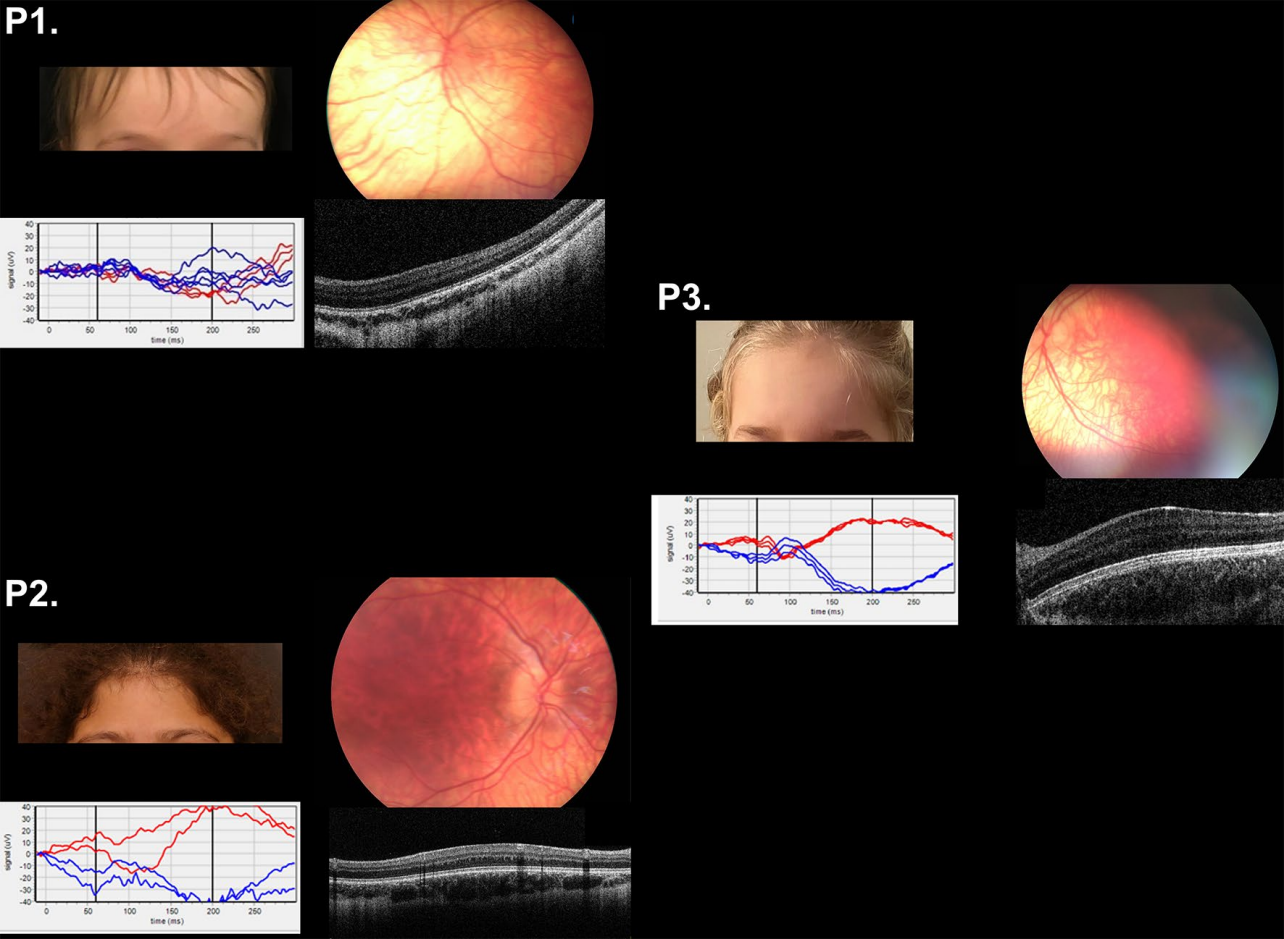
Clinical features of 3 OCA7 patients from the Netherlands, P1 and P2 of Kurdish origin, P3 of Dutch origin. Pigmentation of skin and hair, fundus pigmentation, optical coherence tomography, and visually evoked potentials of patients P1, P2, and P3. None of the patients had iris translucency. Note the normally pigmented skin and hair, which were comparable to family members of all patients. P1, and P3 had fundus hypopigmentation grade 2 with choroid vessels visible in the posterior pole, but not in the macula. Due to poor cooperation it was difficult to capture the macular region in P1, but on fundoscopy the region was clearly pigmented. P2 had grade 1 hypopigmentation of the fundus with choroid vessels visible in the (mid)periphery^1^. In all patients severe foveal hypoplasia (grade 3) was present^7^. Difference signal of the flash visually evoked potentials (VEP) between the electrode on the left hemisphere and the right hemisphere of P2 and P3 show obvious misrouting, in P1 misrouting was present, but less obvious in a poor cooperative 8 months old girl^9^. The red lines are the recordings from the right eye and blue lines from the left eye.

This study was approved by the Medical Ethics Committee of the Leiden University Medical Center and adhered to the tenets of the Declaration of Helsinki. Informed consent was obtained from all participants and/or legal guardians. An additional informed consent was obtained for the publication of the images from the patients in Fig. 2.

We included three patients from two families with two mutations in *LRMDA* from the databases of Bartiméus Diagnostic Center for complex visual disorders. We collected data on all albinism features: pigmentation levels of skin, hair, and eyes (i.e. fundus hypopigmentation and iris translucency), VA, nystagmus, foveal hypoplasia, and misrouting of the optic nerve fibers. Iris translucency and fundus pigmentation were graded^1^. We also graded foveal hypoplasia, according to the scheme of Thomas et al.: in grade 1 a shallow foveal pit is still present, in grade 2 the pit is absent, and in grade 3 and 4 also photoreceptor differentiation is affected^7^. Because the patients were very young, we determined chiasmal misrouting with multichannel flash visually evoked potentials (VEPs) according to ISCEV standards^8^. We calculated the chiasm coefficient from the differential signal of the left hemisphere minus the right hemisphere. To determine if misrouting was present we used the cut-off criteria from Kruijt et al.^9–11^.

We compared the genotype and phenotype of our patients to all patients with two mutations in *LRMDA* and a clinical description in the literature to date.

## Results

Patient data are shown in Table 1. P1 and P2 were members of a consanguineous Kurdish family. P3 was an unrelated Dutch girl. Patients from the Kurdish family had homozygous mutations in *LRMDA*: c.66dupC (p.(Ala23Argfs*39)). The Dutch patient was compound heterozygote: c.566G > A; p.(Gly189Asp) and c.565G > A; p.(Gly189Ser). All three patients had normally pigmented skin and hair comparable to family members, very poor VA (0.7–1.4 logMAR), nystagmus, no iris translucency, grade 1 or 2 fundus hypopigmentation^1^, severe foveal hypoplasia (≥ grade 3)^7^, and misrouting.

**Table 1:** Characteristics of all OCA7 patients.

Subject and etnic background	Variants *LRMDA*	Pigment hair	VA RE/LE*	Nystagmus	Iris translucency	^†^Fundus hypopigmentation	^‡^Foveal hypoplasia	Misrouting
**P1** **Kurdish**	**c.66dupC; c.66dupC**	**Brown**	**1.4 both eyes**	**Yes**	**No**	**Grade 2**	**Yes, grade 3**	**Yes**
**P2** **Kurdish**	**c.66dupC; c.66dupC**	**Brown**	**0.7/0.7**	**Yes**	**No (but grade 1 in first year of life)**	**Grade 1**	**Yes, grade 3**	**Yes**
**P3** **Dutch**	**c.566G > A; c.565G > A**	**Blond**	**0.9/1.0**	**Yes**	**No**	**Grade 2**	**Yes, grade 3**	**Yes**
*P4* *Faroese family*^2^	*c.580C* > *T; c.580C* > *T*	*Red-blond*	*1.0/1.0*	*Yes*	*Yes*	*Grade 1*	*Yes, grade unknown*	*NA*
*P5* *Faroese family*^2^	*c.580C* > *T; c.580C* > *T*	*Blond*	*0.8/0.8*	*Yes*	*Yes*	*Grade 1*	*Yes, grade unknown*	*Yes*
*P6* *Faroese family*^2^	*c.580C* > *T; c.580C* > *T*	*Dark brown*	*0.5/0.5*	*Yes*	*Yes*	*Grade 1*	*Yes, grade unknown*	*Yes*
*P7* *Faroese family*^2^	*c.580C* > *T; c.580C* > *T*	*Blond*	*0.7/0.6*	*Yes*	*Yes*	*Grade 1*	*Yes, grade unknown*	*Yes*
*P8* *Faroese family*^2^	*c.580C* > *T; c.580C* > *T*	*Blond*	*1.3/1.3*	*Yes*	*Yes, only peripheral*	*Grade 1*	*Yes, grade unknown*	*Yes*
*P9* *Faroese family*^2^	*c.580C* > *T; c.580C* > *T*	*Blond*	*0.7/0.7*	*Yes*	*Yes*	*Grade 1*	*Yes, grade unknown*	*Yes*
*P10* *Faroese family*^2^	*c.580C* > *T; c.580C* > *T*	*White*	*1.3/1.3*	*Yes*	*Yes*	*Grade 1*	*Yes, grade unknown*	*Yes*
*P11* *Faroese family*^2^	*c.580C* > *T; c.580C* > *T*	*Dark blond*	*0.8/0.8*	*Yes*	*Yes*	*Grade 1*	*Yes, grade unknown*	*Yes*
*P12* *Lithuanian t*^2^	*c.66dupC; c.66dupC*	*Dark blond*	*0.7/0.7*	*Yes*	*Yes*	*Grade 1*	*Yes, grade 4*	*Yes*
*P14* *Arabian t*^12^	*c.566G* > *A;* *c.566G* > *A;*	*NA*	*Low, NA*	*Yes*	*NA, but ocular hypopigmentation*	*NA, but ocular hypopigmentation*	*Yes, grade unknown*	*NA*
*P14* *French t*^13,14^	*c.89T* > *C; c.163A* > *T*	*Light brown*	*NA*	*Yes*	*Yes*	*Grade unknown*	*Yes, grade unknown*	*NA*
*P15* *Turkish t*^14^	*c.66dupC; c.66dupC*	*Light brown*	*NA*	*Yes*	*NA*	*Ggrade unknown*	*NA*	*NA*
*P16* *Moroccan t*^14^	*c.382C* > *T; c.382C* > *T*	*Light brown*	*0.3/0.4*	*Yes*	*Yes, but discrete*	*NA*	*Yes, grade unknown*	*NA*
*P17* *Turkish patient*^15^	*c.66dupC; c.66dupC*	*Light brown, lighter than family*	*1.0/1.0*	*Yes*	*Yes*	*Grade 1*	*Yes, grade unknown*	*NA*
*P18* *Turkish*^15^	*c.66dupC; c.66dupC*	*Light brown*	*NA*	*Yes*	*Yes*	*Grade unknown*	*Yes, grade unknown*	*NA*

The newly described patients (P1, P2, and P3) are in bold and patients described in the literature are in italics.

In the literature we found four reports with a phenotypical description of in total 15 unique patients with OCA7^2,12–15^. The first report described an inbred Faroese family with 8 affected family members, and one Lithuanian patient^2^. The second study identified one Arabian patient with OCA7^12^. The third study reports on a French, Turkish, and Moroccan patient from France^13,14^, and the fourth paper described two Turkish patients^15^. In total, with our three additional patients, 18 OCA7 patients are now described worldwide. All patients had normal pigmentation of skin and hair compared to family members, all patients showed ocular hypopigmentation: 13/16 had (discrete) iris translucency, and 16/16 had hypopigmentation of the retina. All, had very poor VA median VA 0.8 logMAR (IQR 0.7–1.1), all had nystagmus, and foveal hypoplasia. Misrouting was demonstrated in all patients (11/11) (Table 1).

Khordadpoor-Deilamani et al. also reported on a patient with a homozygous mutation in *LRMDA* (c.267C > A), with an ocular phenotype, but without any further details. Because the patient did not have a clinical description we did not include this patient^16^.

## Discussion

In this study, we describe three newly identified patients with OCA7. We found one novel mutation in a compound heterozygous Dutch girl of non-consanguineous parents: c.565G > A. It predicts the amino acid substitution p.(Gly189Ser) in the LRMDA protein, changing a conserved amino acid. To describe the phenotype of OCA7 we compared our three patients to 15 OCA7 patients described to date. Few patients with OCA7 have been reported in the literature, and most papers report on 1–3 patients only as part of a large cohort of albinism patients, confirming that OCA7 is a rare subtype. All OCA7 patients appear to have a similar, small phenotypic spectrum^1,2,12–15^.

One of the characteristics of albinism is hypopigmentation. Pigmentation in OCA7 can be affected in three different ways: (1) a reduction in DCT as shown in knockdown of the *LRMDA* homolog in zebrafish, (2) lower pH-levels in the melanosomes, and (3) abnormal maturation of melanosomes leading to lack of stage II and III melanosomes and many aberrant melanosomes in OCA7 knockout melanocytes^2,5,6^. Melanosome maturation is also affected in OA1, while in non-syndromic OCA mostly melanin synthesis is impaired but melanosome maturation is normal We hypothesize that maturation of melanosomes plays a more important role in the phenotype of OCA7 in humans than defects in melanin synthesis, because of the remarkable phenotype. None of the OCA7 patients had obvious hypopigmentation of skin and hair compared to siblings or parents. All patients did have hypopigmentation of the eyes, although sometimes only mild. Thus, as in X-linked ocular albinism (OA1), the phenotype seems to be restricted to the eyes. Therefore the term oculocutaneous seems incorrect. Autosomal recessive ocular albinism (AROA) is a term that has been used for patients with albinism with an ocular phenotype caused by mutations in *TYR, OCA2, TYRP, SLC45A2, SLC24A5, or LRMDA* (OCA1-7), but without a clearly defined boundary between calling a phenotype OCA or AROA. To confuse matters even more, children may have a clear OCA phenotype that turns into a more pigmented phenotype later on. This would mean that their condition changes from OCA to AROA during lifetime. Not only the ocular phenotype, but the whole phenotypic spectrum of OCA7 is more comparable to OA1, than to oculocutaneous albinism. OA1 has a smaller phenotypic spectrum than OCA with on average poorer VA and more severe foveal hypoplasia^17^.

Because of the similarities to OA1, we suggest that this type of albinism should be referred to as ocular albinism type 2 (OA2) instead of OCA7.

The severe ocular phenotype and small phenotypic spectrum of OA1 and OCA7 suggest that other factors than (solely) melanin are responsible for normal foveal development and routing of the optic nerve fibers. Probably, the defect in OA1 and OCA7 lies more downstream in the pigmentation pathway, without a rescue that may occur in other type of albinism. Intriguingly, Bakker et al. could not detect *LRMDA* expression in embryonic RPE of seven weeks, nor in their own retinal organoid dataset^18^. Further investigation of the function and role of *LRMDA* in the retinal pigmentation pathway will probably help in unravelling the responsible factors for the combination of foveal hypoplasia and misrouting of the retinal ganglion axons.

In conclusion, OCA7 has a severe ocular phenotype but normal skin and hair pigmentation, comparable to OA1. As hypopigmentation is restricted to the eyes, we suggest to rename this type of albinism: ocular albinism type 2 (OA2).

## Data Availability

All data that are analysed during this study are included in this published article not included in this published article, other patient information are available from the corresponding author on request.
